# Visible Light-Induced Antibacterial Activity of Pigments Extracted from Dregs of Green and Black Teas

**DOI:** 10.1155/2021/5524468

**Published:** 2021-06-12

**Authors:** Renny Indrawati, Elok Zubaidah, Aji Sutrisno, Leenawaty Limantara, Melisa Megawati Yusuf, Tatas Hardo Panintingjati Brotosudarmo

**Affiliations:** ^1^Department of Food Science and Technology, Faculty of Agricultural Technology, Universitas Brawijaya, Malang 65145, Indonesia; ^2^Ma Chung Research Center for Photosynthetic Pigments, Universitas Ma Chung, Malang 65151, Indonesia; ^3^Chemistry Study Program, Faculty of Science and Technology, Universitas Ma Chung, Malang 65151, Indonesia; ^4^Center for Urban Studies, Universitas Pembangunan Jaya, South Tangerang 15413, Indonesia

## Abstract

Chlorophyll and its derivatives are potential natural sensitizers frequently applied in antimicrobial photodynamic therapy. Chlorophyll derivatives are formed naturally during tea processing, but they do not contribute to the color of tea infusions and thus are presumably left in the tea dregs. The present study aimed to investigate (i) the chlorophyll remnants in the pigments recovered from dregs of green and black teas and (ii) the antibacterial activity of pigments extracted from the tea dregs upon illumination using a light-emitting diode (LED) as the light source. Pigment analysis using high-performance liquid chromatography (HPLC) revealed the presence of main degradation products of chlorophylls, such as pheophytin and its epimers, pyropheophytin, and pheophorbides. In vitro assays demonstrated significant reductions in the number of viable bacteria in the presence of the pigments after 30 min of incubation with LED light irradiation. The descending order of bacterial susceptibility was *Listeria monocytogenes* > *Staphylococcus aureus* > *Escherichia coli* > *Salmonella typhi*. At an equivalent irradiation intensity, the blue and red LEDs could stimulate a comparable inactivation effect through photodynamic reactions. These findings demonstrated the valorization potential of tea dregs as a source of chlorophyll derivatives with visible light-induced antibacterial activity.

## 1. Introduction

In the midst of escalating public health concern over the global-scale spread of infectious diseases, antibiotic resistance, and issues related to energy crises and environmental problems, the task to combat microbial contamination has become increasingly challenging. Recently, much research effort has been focused on the development of photodynamic inactivation technology that combines physical and chemical reactions to induce the death of targeted microorganisms, specifically antimicrobial photodynamic therapy as a promising nonthermal disinfection technology. This novel therapy has been reported to offer significant breakthroughs such as rapid inactivation of pathogenic microorganisms, effective elimination of the resistant strains, and even the biofilm-producing bacteria, which are often resistant to treatments [1–3].

The three components in a typical photodynamic reaction are a photosensitizer, light, and oxygen. Photosensitizers are substances that can capture photon energy from the light and transfer them to other surrounding molecules. Energy released from the photosensitizer causes the formation of radicals from sensitive surrounding molecules, such as proteins, lipids, and oxygen [4]. Radical oxygen species (ROS) are free radicals that are highly reactive and cytotoxic; hence, prompt cell death is triggered by excessive concentration of ROS. Chlorophylls (particularly some of their derivatives) have been reported as promising candidates for natural sensitizers owing to their unique photophysical and photochemical properties [5, 6]. They are characterized by strong absorption of light in the blue and red regions of the visible spectrum [7], offering the significant benefit of harnessing the energy of light that is harmless and, therefore, the preferred sensitizers for the application of photodynamic inactivation [8]. Moreover, light-emitting diodes (LEDs) can be used as economically feasible photon sources [9].

**Figure 1:**
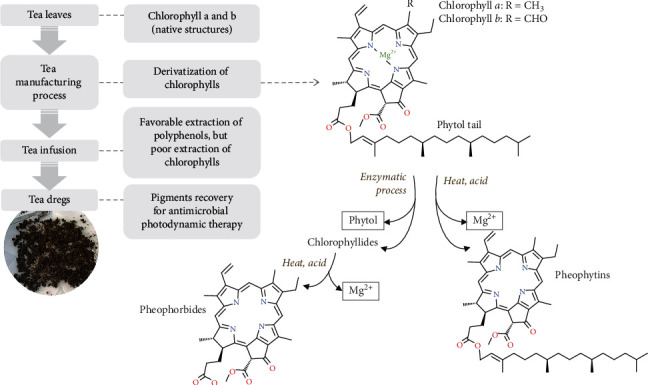
Schematic diagram of the research background. The manufacturing process of tea leads to derivatization of naturally occurring chlorophylls in the tea leaves. The derivatives (pheophytins and pheophorbides) are known as potential candidates of photosensitizers for antimicrobial photodynamic therapy. Since these pigments are poorly extracted in water, they could be recovered from the tea dregs for further utilization.

To date, many studies have highlighted the efficacy of photodynamic inactivation of microorganisms using semisynthetic chlorophyll derivatives, such as chlorophyllin sodium salt, pheophorbide, pheophytin, and chlorin e6 [10–14]. The natural structure of chlorophyll is susceptible to degradation due to the alterations in environmental pH and temperature, which leads to the release of central magnesium ion, or the breakdown of the phytol tail [15] (see Figure 1). Interestingly, such degradation also occurs during the tea processing and is unique according to the types of tea. Suzuki and Shioi [16] reported the detection of various chlorophyll derivatives in tea. Considering the fact that these compound groups are poorly extracted during tea infusion using hot water [17], pigment recovery from the tea dregs might provide an alternative source of chlorophyll derivatives as well as possible valorization of used tea leaves.

Therefore, the objectives of this study were (i) to perform the analysis of remnant chlorophylls and their derivatives using pigment recovered from the dregs of green and black teas and (ii) to evaluate the potency of the pigments as sensitizers for antimicrobial photodynamic therapy under LED light exposure. Pigment analysis and characterization were carried out using a UV-visible spectrophotometer and high-performance liquid chromatography (HPLC) with a diode array detector. Thereafter, in vitro microbial assays were conducted under blue and red LED illumination. Among the diverse microbial groups, pathogenic bacteria are the main etiological agents posing the highest threat to the public health [18]; hence, four major food-borne bacteria were used as indicators in this study, namely, *Listeria monocytogenes*, *Staphylococcus aureus*, *Escherichia coli*, and *Salmonella typhi*.

The hypothesis of the present study was that “the red and blue light irradiation on the pigments extracted from infusion dregs of green and black tea possess antimicrobial effect on pathogenic bacteria.” The hypothesis was further divided into three subhypotheses: (i) the red light irradiation on the pigments extracted from dregs of green and black teas have an antimicrobial effect on pathogenic bacteria, (ii) each pathogenic bacterium shows different sensitivity toward photodynamic inactivation using pigments irradiated with red light, and (iii) the irradiation of red and blue light on the pigments causes different inactivation effects on pathogenic bacteria.

## 2. Materials and Methods

### 2.1. Pigment Extraction and Analysis

#### 2.1.1. Preparation of Tea Dregs and Pigment Recovery

Commercial green and black teas were obtained from a tea factory (PT Sari Melati Sejahtera, Pekalongan, Indonesia). The samples were brewed in hot water (90°C) for 5 and 20 min at a ratio of 1 : 100 (tea to water). The tea dregs were subsequently separated, spread onto filter papers, and dried at ambient temperature overnight to reduce its water content. Thereafter, dregs was mixed with acetone at a ratio of 1.5 : 10 (solid to solvent) and vortexed, and the supernatant was collected after centrifugation (16,000 × g, 1 min). This extraction procedure was repeated three times until the dregs turned pale. The solvent was then removed through vacuum evaporation, and the extracted pigment was collected in a vial and kept at a low temperature (−20°C) until further analysis.

#### 2.1.2. Pigment Analysis

The concentrations of total chlorophylls derivatives were calculated according to the equation of Lichtenthaler [19] using a UV-visible spectrophotometer (UV-1800, Shimadzu, Kyoto, Japan). Pigment composition was determined using HPLC according to a previously published method [20]. Each sample of extracted pigment was dissolved in acetone and filtered (0.22 *μ*m, Nylon, Whatman, Kent, UK) prior to injection into a C_8_ column installed on an HPLC instrument (LC-20A) that has been equipped with an SPD-20MA diode array detector (Shimadzu, Kyoto, Japan). Each separated peak in the visible range (350–700 nm) was identified based on its retention time and spectral characteristics, by comparison to [16, 20]. The entire extraction and analysis procedures were performed under dim light to avoid pigment degradation.

### 2.2. Pigment Isolation

A single pigment was used as a positive control during the photodynamic experiments. Chlorophyll a (Chl a) and b (Chl b) were isolated from the leaves of *Pleomele angustifolia*, which are known to have a high content of chlorophyll. The extracted crude pigment was first dissolved in acetone and separated from carotenoid fractions using column chromatography with silica gel 60 as the stationary phase and hexane-acetone as the mobile phase. The chlorophylls were purified and collected after elution in HPLC with a gradient mobile phase comprising methanol and acetone [21]. Pheophytin a (Pheo a) was obtained from the pure chlorophyll a after reacting with the acetic acid in acetone, and further elution of pigments using the HPLC was performed according to an established method [20].

### 2.3. Photodynamic Experiments

#### 2.3.1. Preparation of Sensitizers

The concentration of each isolated pigment was firstly adjusted to 100 *μ*g·mL^−1^ in organic solvent based on the Beer–Lambert equation with a defined molar extinction coefficient for each chlorophyll a (78.75 × 10^3^ Lmol^−1^·cm^−1^ at 663 nm in acetone), chlorophyll b (56.26 × 10^3^ Lmol^−1^·cm^−1^ at 643 nm in diethyl ether), and pheophytin a (46.50 × 10^3^ Lmol^−1^·cm^−1^ at 667 nm in acetone) [22, 23]. Although pigments extracted from tea dregs consisted of various chlorophyll derivatives, they exhibited maximum absorption at 667 nm, indicating the abundance of pheophytin a. Thus, the concentration was first determined by adjusting the absorbance intensity at 667 nm for pheophytin a, and total pheophytins were confirmed by using the formula of Lichtenthaler [19]. After complete evaporation of the solvent, the dried pigment was dispersed in an identical volume of sterile 1% Tween 80 (w/v) using sonication. The surfactants were used to prevent any molecular aggregation of chlorophylls, which may inhibit their bioactivity as sensitizers [12]. The same aqueous surfactant solution was used as a negative control in the photodynamic experiments.

#### 2.3.2. LED Lamps

**Figure 2:**
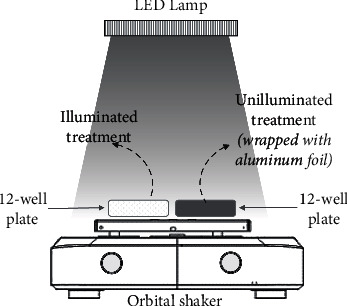
Schematic illustration for the set-up of photodynamic experiment. The suspensions containing sensitizer and liquid culture of bacteria were transferred onto 12-well plate and positioned on the orbital shaker (100 rpm, 30 min, at 18 ± 3°C) under the LED lamp (65.2 Wm^−2^). The equivalent samples for unilluminated treatment were put in an identical plate wrapped with aluminum foil.

Identical red and blue LED lamps (Yomiko YL-2550, 50 Watt, 4800 Lumens) were used as the light sources in the photodynamic treatment. The emission spectrum of each LED lamp was recorded using a spectrometer (Ocean Optics USB4000, FL, USA). The LED lamps were assembled and set above the orbital shakers to give 300 ± 15 *μ*mol·m^2^s^−1^ photons (measured using Apogee Instruments MQ-200, USA, equal to approximately 65.2 Wm^−2^) on the well plate (see Figure 2). The estimated photon energy of the blue light (445 nm) and red light (640 nm) was 2.79 and 1.94 eV, respectively.

#### 2.3.3. Microbial Assay

Four pathogenic bacteria were used as targeted indicators, comprising two Gram-positive and two Gram-negative strains. *Listeria monocytogenes* (FNCC 0156) was obtained from the Food and Nutrition Culture Collection Division, Gadjah Mada University, Jogjakarta, whereas *Staphylococcus aureus* (ATCC 6538), *Escherichia coli* (ATCC 8739), and *Salmonella typhi* (clinical isolate) were acquired from the Department of Pharmacy, Widya Mandala Catholic University, Surabaya, Indonesia. The microbial assay consisted of two stages: (1) preliminary screening of antimicrobial activity using a red LED; (2) the comparative inactivation capacity of the treatments using blue and red LEDs.

Briefly, an inoculum of 10^8^ CFU·mL^−1^ was prepared in nutrient broth (NB), and a volume of 75 *μ*L was transferred to an Eppendorf tube containing 1,425 *μ*L of the sensitizer in 1% Tween 80. The suspension was then aliquoted into individual 400 *μ*L volumes and transferred into the wells on a 12-well plate for LED exposure, each for illuminated and unilluminated samples. The plates were then placed on a bench top orbital shaker (MaxQ 2000, Thermo Scientific, Iowa, USA) with gentle agitation (100 rpm) and illuminated for 30 min at room temperature (18 ± 3°C). After the illumination, each sample was immediately diluted with 4.6 mL Mueller–Hinton Broth (MHB), and 0.5 mL of the suspension was added to 9.5 mL of peptone solution. After vortexing, 20 *μ*L of the inoculum was plated in Trypticase Soy Agar (TSA) and incubated for 24 h at 34°C, and CFU number was counted as total viable cells. The percentage of inactivation was calculated based on the difference in total viable cells found in samples with and without illumination (*L* and *D*, respectively), using the formula ((*D* − *L*) × 100/*D*). All microbial experiments were performed in triplicate.

#### 2.3.4. Photostability Study

A photostability study was carried out by dissolving the pigments in acetone and illuminating them continuously with LEDs for 60 min at room temperature. The evolution of the electronic absorption spectrum was recorded periodically using a UV-visible spectrophotometer (UV-1700, Shimadzu, Kyoto, Japan) and examined for any decrease or shift in the absorption peaks or shoulders. Since remnant of plant carotenes might be present, the photostability of pigments was also evaluated using *β*-carotene as standard reference (Chameleon Reagent, Osaka, Japan).

### 2.4. Statistical Analysis

The results were reported as the mean ± standard error of the mean (M ± SEM) from three experimental replicates. Comparison of treatments was performed using one-way analysis of variance (ANOVA) at a significance level of 0.05, subsequently followed by either Fisher's least significant difference test or Dunnett's test for comparison to the control group. All statistical analyses were performed using the Minitab software ver. 17.00 for Windows.

## 3. Results and Discussion

### 3.1. Chlorophyll Derivatives in the Pigment Extracted from Tea Dregs

Hot water extraction yielded tea dregs in a dense color, indicating the presence of residual pigments. According to previous studies by Suzuki and Shioi [16] as well as Donlao and Ogawa [17], only 2% of chlorophyll a or 7% of total chlorophylls from a tea were detected in the tea infusion. Nevertheless, there is limited information on the remnant pigments in the tea dregs. To the best of our knowledge, the first and only report was provided by Higashi-Okai et al. [24], who used the conventional thin-layer chromatographic method to separate pigments in Japanese green tea after hot water extraction.

**Figure 3:**
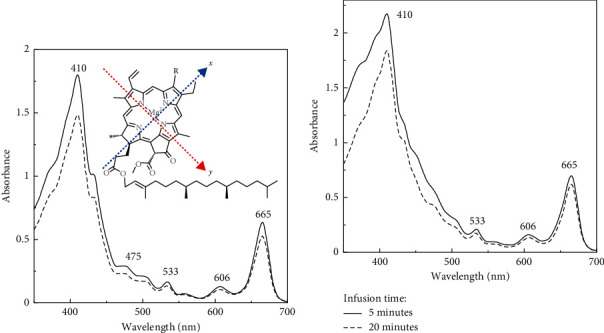
The absorption spectra of the pigments extracted from tea dregs of green (a) and black teas (b) after 5 and 20 min of hot water infusion at 90°C. Figure 3(a) shows the diagonal arrows for the direction of two polarization axes in the structure of chlorophyll, *x* and *y*, contributing to the absorption band at 665 nm (Qy band), 533 (Qx band), and 410 nm (overlapped Soret band).

In the present study, we found that the extraction of tea dregs using acetone resulted in a pale pellet, suggesting the attainment of pigment recovery. The pigments extracted from both green and black tea dregs exhibited comparable absorption spectra (see Figure 3), with two obvious maxima at 410 and 665 nm, with several weak absorption signals at 475, 533, and 606 nm. The typical absorption spectra of the chlorophyll groups showed along the *x*- and *y*-axis electronic transitions within the pigment molecules (Figure 3(a)), with the weak Qx band near 550 nm and strong Qy band near 660 nm, whereas the intense Soret band in the blue region indicated overlapping region [25]. Chlorophyll a and b are characterized by distinctive Soret bands at 430 and 457 nm, with peculiar ratios of intensity at Soret: Qy bands of 1.23 and 2.82, respectively [26]. Their molecular derivatization causes Soret to shift to higher energy (blue shift) as well as an increase in the Soret to Qy band intensity ratio. In fact, chlorophyll a is three to four times more abundant than chlorophyll b [27]; therefore, the spectrum of chlorophyll a often masks that of chlorophyll b. In accordance with the absorption spectra of the pigments, the observed Soret band at 410 nm and the increased Soret to Qy band ratio (2.8 in Figures 3(a) and 3.1 in Figure 3(b)) indicated the dominant chlorophyll derivatives in pigments extracted from tea dregs.

Moreover, the absorption intensity of the pigments reduced after prolonged brewing time of 20 min, although without any significant alteration of the absorption pattern. The reductions of intensity at 665 nm (Qy band) were 17.6 and 12.4% for green and black teas, respectively. This reduction could be attributed to the extraction of saponins from tea due to prolonged brewing time of about 25–30 min [28]. Although saponins are known as weak surfactants, traces of this compound could enhance the solubility of hydrophobic pigments in tea infusion [16].

**Figure 4:**
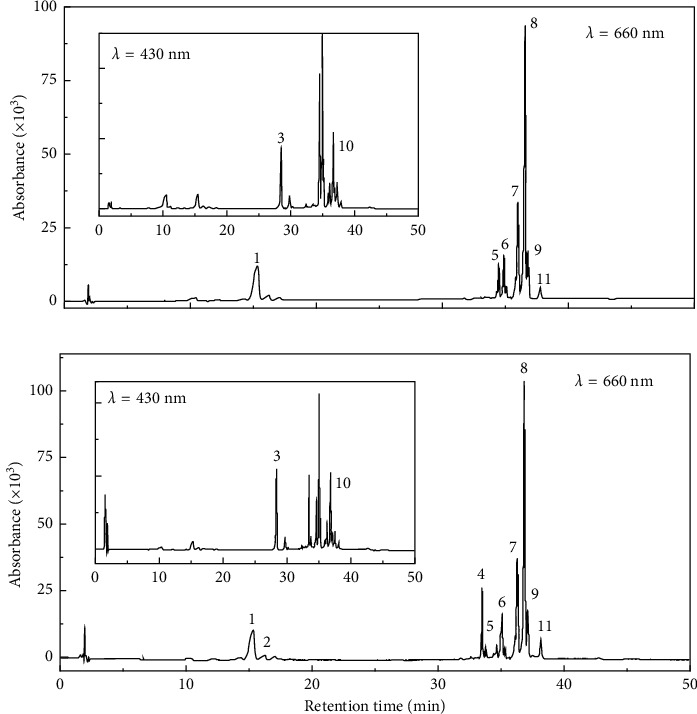
HPLC profiles for pigments extracted from dregs of green (a) and black (b) teas, primarily detected at 660 nm for chlorophyll groups and 430 nm (inset) for carotenoids. The peak number corresponds to the identified fractions as listed in Table 1.

HPLC analyses were performed to determine the fractions of the chlorophyll derivatives.  Figure 4 shows the chromatograms for the pigment recovered from the dregs of green and black teas. The chromatograms were monitored primarily at 660 nm for the detection of chlorophyll groups and at 430 nm for carotenoids. Each distinct peak was identified according to the specific maxima of [16], as listed in Table 1. Nine species of chlorophylls were detected, most of which belonged to chlorophyll derivatives, except chlorophyll b, which was only observed in moderate amount for black tea. Two species of carotenoids were identified as *β*-carotene and lutein, known as the accessory photosynthetic pigments exhibiting light absorption at approximately 450 and 475 nm, respectively.

The absence of chlorophyll a in the pigments signified complete conversion of chlorophyll a into its derivatives, manifested as pheophytin a and pheophorbide a, as well as their epimers and/or the fractions having adjacent peaks due to structural similarity. The possible species for pheophytin a could be hydroxy-pheophytin a (peak 7), which was eluted before pheophytin a, as well as its less-polar epimer (pheophytin a', peak 9) and pyropheophytin (peak 11), which were eluted after pheophytin a [29]. On the contrary, the remnant of chlorophyll b was still observed in pigments recovered from the dregs of black tea, accompanied by its pheophytins. This could be attributed to the earlier conversion of chlorophyll b in green tea compared to black tea, as evidenced by intense signals of pheophytin b species (peaks 5 and 6). Chlorophyll a is known to be more sensitive and susceptible to acid or heat, and it releases its central magnesium ion up to nine times faster than chlorophyll b [30].

During tea leaves processing, heat treatment methods such as steaming (Japanese style) or frying (Chinese style) are commonly used to for green tea production, whereas black tea processing involved fermentation which allows advanced oxidation in the presence of polyphenol oxidase [31]. Thus, the temperature and time of the thermal treatment in green tea would determine the derivatization efficiency of chlorophyll a and b [17, 32]. Thermal treatment hastens the degradation of both chlorophylls, while the fermentation of black tea at lower temperature has less effect on the degradation of chlorophyll b. A similar result was reported by Wijaya et al. [33], who compared chlorophyll fractions in green and black teas based on peak area in chromatograms. However, based on quantitative analysis, total pheophytins recovered from the dregs of green and black teas were 3.08 ± 0.18 and 2.31 ± 0.31 mgg^−1^ (dry weight), respectively. The production process of black tea is longer than that of green tea; therefore, further degradation of chlorophyll derivatives into colorless compounds was possible.

### 3.2. Light-Induced Antibacterial Activity of Pigments Extracted from Tea Dregs

The use of light energy in microbial control has been extensively studied and applied, mainly in the range of ultraviolet (200–400 nm) and blue light (400–470 nm) [1]. However, the application of photodynamic treatment to kill tumor cells requires the wavelength within the therapeutic range (600–800 nm) which has less damaging effects on the host tissue [34]. The low-energy red light irradiation is less effective in deactivation of pathogens [35]. Thus, in the present study, a preliminary investigation of visible light-induced antimicrobial activity in the pigments extracted from tea dregs was conducted using a red LED lamp with a peak emission wavelength of 640 nm, to stimulate the chlorophyll compounds at the Qy absorption band.

**Figure 5:**
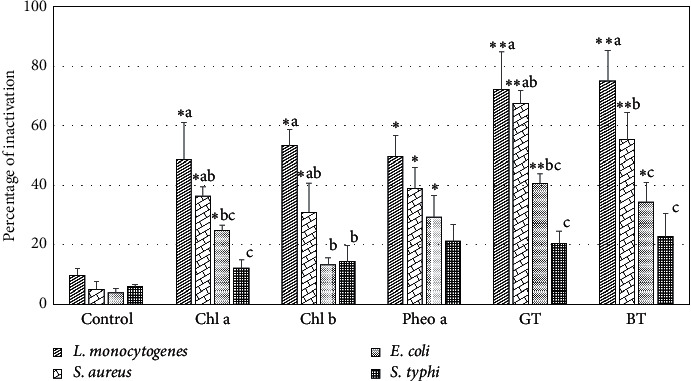
Percentage of bacterial inactivation after incubation of single broth cultures with various sensitizers under illumination of red LED for 30 min. The types of applied sensitizers were as follows: sterile 1% Tween 80 as the medium for pigments dispersion (control), chlorophyll a (Chl a), chlorophyll b (Chl b), pheophytin a (Pheo a), and pigments extracted from the dregs of green tea (GT) and black tea (BT). The error bars represent standard error of the mean for three repetitions in each treatment. The upper symbols denote statistical significance based on treatment to control comparison using Dunnett's test at confidence level of 0.95 (^*∗*^) and 0.99 (^∗∗^), whereas the different letters indicate significant differences (*p* < 0.05) among bacterial species in the same group of sensitizers.

Photodynamic reactions take place when the sensitizer absorbs photons which undergo decay, resulting in intramolecular energy transfer, followed by photooxidation by singlet oxygen and radicals. In a relatively short time, the radical species can be generated from both environmental oxygen and biomolecules (lipids and proteins) present in the cell membranes, causing cytotoxic effects and cell membrane damage. Here, we demonstrated that after 30 min of incubation, the number of viable cells in the illuminated treatments was reduced compared to that in the dark condition. The difference between total viable cells in the dark and illuminated treatment was quantified as the percentage of inactivation caused by photodynamic reaction in the four indicator bacteria (Figure 5).

The percentage of inactivation was significantly higher (*p* < 0.05) in the experimental groups supplemented with chlorophyll compounds and pigments, compared to the negative control, indicating the occurrence of photodynamic inactivation, and hence the first subhypothesis was accepted. Meanwhile, the control group also demonstrated significant inactivation of indicator bacteria. The aqueous surfactant solution (1% w/v Tween 80) was used to maintain the pigments as monomers at low concentrations, without exhibiting any cytotoxic effect [12]. The reduction in total viable cells found in the control group could be caused by the presence of an endogenous sensitizer. Several metabolic products of bacteria have been identified as potential endogenous photosensitizers that augment their sensitivity to light irradiation, such as protoporphyrin IX, coproporphyrin III, and uroporphyrin, depending on the bacterial strains [36, 37]. Mild inhibition in the growth of *E. coli* and *S. aureus* following treatment using 630 nm light has been reported by Nussbaum et al. [38]. Therefore, the presence of exogenous photosensitizers could have contributed to the inactivation capacity of the LED light.

The percentages of inactivation in the bacteria cultures treated with the pigments extracted from tea dregs were comparable to those of the positive control (single chlorophyll compound) and significantly higher than those of the negative control (*p* < 0.01), particularly for *L. monocytogenes*, *S. aureus*, and *E. coli*. This finding indicates the potency of the tea dregs as a source of sensitizers without further pigment purification. The combination of two or more sensitizers could enhance the synergistic effect and increase the capacity of photodynamic inactivation [39].

Figure 5 also illustrates the significant difference in the inactivation effect between the indicator bacteria (*p* < 0.05); hence, the second subhypothesis was accepted. The descending order of sensitivity toward photodynamic inactivation was *L. monocytogenes* > *S. aureus* > *E. coli* > *S. typhi*. The greatest photodynamic impact was observed for *L. monocytogenes*, ranging from 48.55 to 74.92% of cell death. The lowest photodynamic inactivation was observed for *S. typhi*, ranging from 12.00 to 22.76% of cell death. Gram-positive bacteria (*L. monocytogenes* and *S. aureus*) generally exhibited higher susceptibility to antimicrobial photodynamic treatment compared to Gram-negative bacteria (*E. coli* and *S. typhi*). Most Gram-positive bacteria have a single cell wall comprising peptidoglycan with teichuronic and lipoteichoic acids, hence a fairly high degree of porosity, whereas Gram-negative bacteria, in addition to the peptidoglycan layer, possess a highly organized outer membrane comprising lipopolysaccharides, phospholipids, lipoproteins, and proteins [5]. The complexity and robustness of the cell wall structure are the determining factors for attachment and absorption of the sensitizers.

### 3.3. Effect of Wavelength of LED Light on Photodynamic Inactivation Using Pigment Extracts from Tea Dregs as Sensitizers

Based on the absorption patterns of pigments extracted from the tea dregs (Figure 3), there are two maxima in the blue and red regions. Several studies on antimicrobial photodynamic therapy preferred to use light with shorter wavelengths owing to its higher energy [40–42], while some studies reported the effectiveness of red light, although the absorption intensity of chlorophyll compounds in this region was not as high as that in the blue region [12, 43]. To date, information on parallel comparison of photodynamic inactivation using light at both wavelengths is scarce.

**Figure 6:**
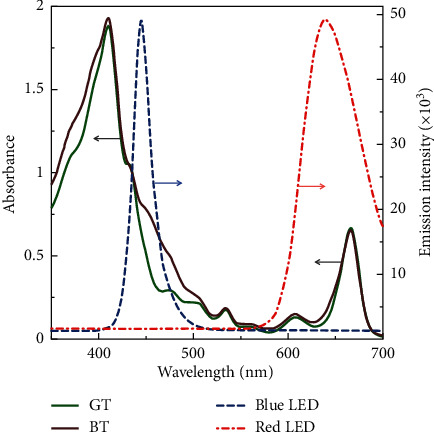
Absorption spectra of pigments extracted from the dregs of green (GT) and black teas (BT), being overlapped with the blue and red LED emission spectra. The arrows point to the axis for the marked curves.

In the present study, the blue and red LED exhibited emission at 445 ± 50 and 640 ± 60 nm, respectively.  Figure 6 shows the emission spectra of the LEDs which are overlapped with the absorption spectra of pigments recovered from the dregs of green and black teas. The Soret absorption band intersects the emission region of the blue LED, whereas the Qy band coincides with the emission region of the red LED. The total number of photons administered to the samples in the well plate was equalized by adjusting the distance between the lamps and the well plate.

**Figure 7:**
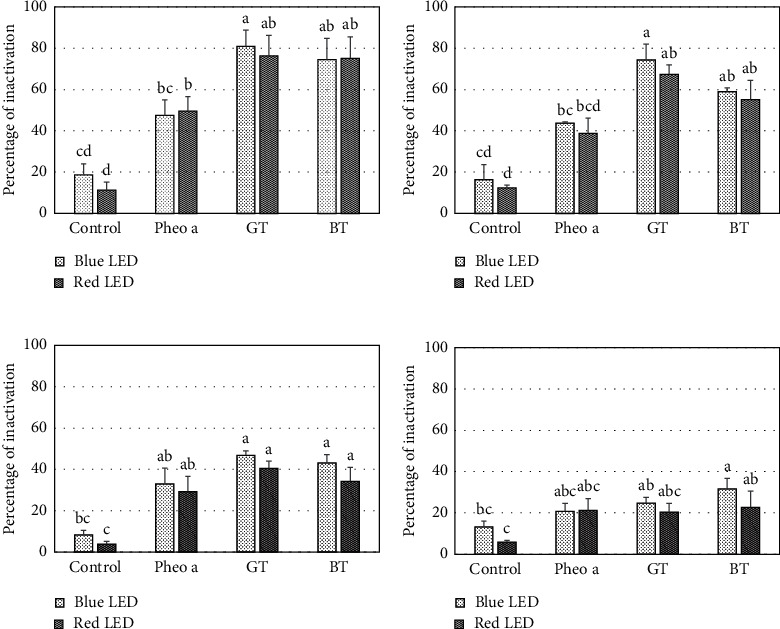
Percentages of inactivation observed in four species of bacteria: (a) *Listeria monocytogenes*, (b) *Staphylococcus aureus*, (c) *Escherichia coli*, and (d) *Salmonella typhi*, by using the treatment with various sensitizers and under illumination of blue and red LEDs for 30 min. The types of applied sensitizers were as follows: sterile 1% Tween 80 as the medium for pigments dispersion (control), pheophytin a (Pheo a), and pigments recovered from the dregs of green tea (GT) and black tea (BT). The error bars represent standard error of the mean for three repetitions in each treatment. The upper letters indicate the significant differences (*p* < 0.05) among treatments according to pairwise Fisher's test.

Figure 7 shows the percentages of inactivation in the cultures of indicator bacteria after photodynamic treatment under blue and red LEDs. Pheophytin a (Pheo a), as the dominant pigment of both teas, was selected as the positive control. There was no significant difference in the variation of LED wavelengths on treatment effect (*p* > 0.05) for all bacteria, showing that both LEDs could be used to induce comparable inactivation effects. Thus, the third subhypothesis was rejected.

Although percentage of inactivation was higher in the treatment using blue LED compared to that in red LED, the difference was not statistically significant. This finding could be attributed to the difference in photon energy and the absorption profile of the sensitizer. The absorption intensity of the sensitizers in the blue region was consistently higher than that in the red region, suggesting the augmenting effects attributed to the absorption properties of the added exogenous sensitizers and the potential endogenous sensitizers (porphyrins group).

As reported by Ghate et al. [35], the use of an LED with 461 nm wavelength at an irradiation intensity of 221 Wm^−2^ on the cultures of *L. monocytogenes* and *S. aureus*, resulted in reduction effects of 5.2 and 4.7 log CFU·mL^−1^, respectively. This treatment effect was achieved by applying high-dose blue LED irradiation for 7.5 h without exogenous photosensitizer and combined with chilling treatment (10°C). This method was shown to effectively reduce the total viable cells of psychrotrophic and mesophilic bacteria. In the present study, LED lights of lower irradiation intensity (65.2 Wm^−2^) were used in combination with the pigments recovered from the tea dregs as sensitizers, and after 30 min incubation at room temperature (18 ± 3°C), reductions of *L. monocytogenes* and *S. aureus* were up to 2.78 and 1.98 log CFU·mL^−1^, respectively. In addition to the types of bacteria, the efficiency of antibacterial photodynamic therapy can be enhanced by controlling the concentration of photosensitizer, incubation time, and irradiation dosage [44]. Since nonselective media were used for cell enumeration purpose in the present study, the number of surviving cells counted could have included the possible sublethal injured cells. In addition, since photodynamic inactivation process happens very quickly and effectively, the risk of bacterial resistance due to the treatment is highly unlikely [45, 46].

**Figure 8:**
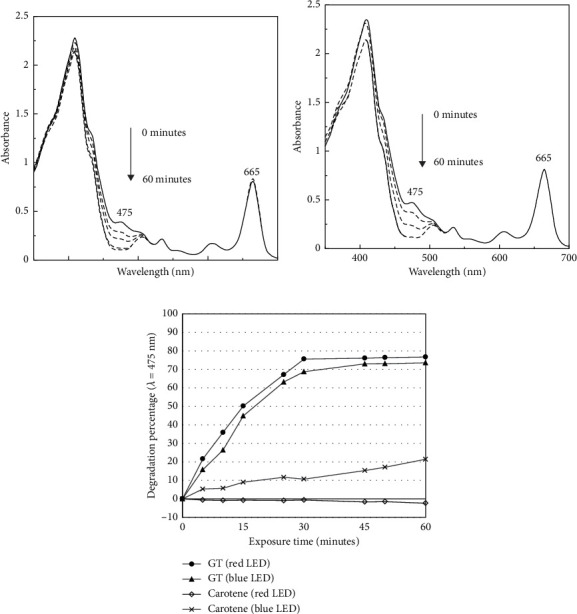
Absorption spectra of pigments recovered from the dregs of green tea (GT) in acetone solvent during irradiation treatment 0–60 min using blue (a) and red (b) LEDs. The observed reductions in absorption at 475 nm were plotted and compared to the equivalent treatment for standard carotene (c).

A photostability experiment was carried out to predict the molecular dynamics during blue and red light irradiation. Figures 8(a) and 8(b) depicts the spectral evolution of the absorption of pigments recovered from the dregs of green tea, at an illumination duration of 0 to 60 min and at the same irradiation intensity. There was no significant change in the absorption at the Qy band (665 nm), revealing the stability of chlorophyll derivatives even up to 60 min of LED illumination. Chlorophyll derivatives, such as pheophytins and pheophorbides, are known to have favorable photostability; hence, they have been recognized as better candidates as photosensitizers compared to native chlorophylls which have central magnesium ions [12, 47]. A sensitizer with high photostability indicates its high ability to return to original state once the photosensitization reaction is complete, and it could continue to generate more radicals in the subsequent cycles of photosensitization.

The gradually diminished absorption intensity at approximately 475 nm resembled the light absorption region of carotenoids. This obvious degradation of carotenoids in the mixture of light-treated chlorophylls could be due the effect of photodynamic treatment. When the same illumination treatment was applied to the standard carotene, the effect of degradation was reduced (Figure 8(c)). A photodynamic reaction between the photons with chlorophylls compounds and environmental oxygen occurred when recovered pigments were exposed to blue or red LED, thus giving rise to free radicals. Carotenoids are particularly sensitive to free radicals due to their protective role in the photosynthetic light-harvesting complex. If carotenoids are present in a photodynamic system with a photostable sensitizer, an adequate number of radicals will be produced through photodynamic reactions, causing the carotenoids to undergo rapid oxygenations. Such an exhaustive process leads to the conversion of carotenoids into smaller and colorless end products. The same phenomenon was reported by Fiedor et al. in a photodynamic system having both bacteriopheophytin and carotenoid [48, 49].

Figure 8(c) further revealed that the rate of degradation upon irradiation on standard carotene was higher when the blue LED was applied. This is an important finding to guide the decision making process on the application of light-induced antibacterial agents. Blue (460 nm) LED has been previously used to eliminate *Salmonella* in orange juice, but an undesirable change in product color was observed due to carotenoid degradation [50]. In contrast, the use of red LEDs was preferred in the handling of fresh produce. The illumination of red LEDs (630–660 nm) on lettuce and broccoli during postharvest storage was found to better preserve the chlorophyll and ascorbic acid contents as well as the food color, compared to that of blue LEDs (450–470 nm) [51, 52]. Therefore, since we found no significant difference in the use of blue and red lights to induce the light-induced antibacterial activity using pigments recovered from tea dregs, light with longer wavelength and lower energy should be preferred.

Nevertheless, the wavelength and intensity of the light source contribute to the dosimetry of photodynamic treatment by influencing the rate of photobleaching of the sensitizers. When incubation time is prolonged, the generated singlet oxygen also mediates the photodegradation of the sensitizer, which leads to color fading due to molecular fragmentation [53, 54]. Both photodynamic and photobleaching reactions typically occur during illumination; hence, the optimum set-up for an ideal photodynamic inactivation system should be further investigated. In perfecting the application of the photodynamic principle in decontamination procedure using pigments from tea dregs, the reversible photobleaching nature of chlorophylls becomes an advantage. Color fading of sensitizers could be used as an indicator to halt a photodynamic treatment. To date, there is no report on the toxicity of colorless degraded products of chlorophylls, and the same pathway also occurs in the catabolism of chlorophylls in green plants [55, 56]. Moreover, according to previous fruit decontamination study conducted by Buchovec et al. [41], radicals generated from photodynamic reactions typically have a very short half-life, and the risk of inducing additional free radicals in the food matrix was negligible.

## 4. Conclusions

Chromatographic and spectrophotometric analyses confirmed the presence of chlorophyll derivatives in the dregs of green and black teas, and the total pheophytins recovered using acetone were 3.08 ± 0.18 and 2.31 ± 0.31 mgg^−1^ (dry weight), respectively. The illumination treatment on the pigments, using LED light at an irradiation dosage of 65.2 Wm^−2^, exhibited antibacterial effect with comparable inactivation capacity of a single chlorophyll compound at a concentration of 100 *μ*g·mL^−1^. Gram-positive bacteria (*L. monocytogenes* and *S. aureus*) were more susceptible to photodynamic inactivation than Gram-negative species (*E. coli* and *S. typhi*). A significant reduction of *L. monocytogenes* and *S. aureus* up to 72.14% (2.78 log CFU·mL^−1^) and 67.38% (1.98 log CFU·mL^−1^), respectively, was achieved after 30 min of illumination treatment at room temperature. The blue and red LEDs at an equivalent irradiation intensity showed no significant difference in the inactivation effect on the four indicator bacteria using the pigments recovered from tea dregs as photosensitizers. However, for the purpose of food decontamination, red LED was shown to cause less degradation effect on the endogenous pigments in the food matrix. These findings revealed the potential utilization of the tea dregs as sensitizers and a foreseeable future of applying light-induced antibacterial agents from natural resources for microbial decontamination purposes.

## Figures and Tables

**Table 1:** Detection of chlorophyll and carotenoid fractions found in the pigments extracted from dregs of green and black teas. The peak numbers correspond to the identified peaks in the chromatograms of Figure 4.

Peak no.	Retention time (min)	Fractions	*λ* max (nm)				
1	15.8	Pheophorbide a	409	508	538	609	665
2^*∗*^	16.6	Pheophorbide a sp.	409	—	—	—	665
3	28.4	Lutein	447	—	—	—	475
4^*∗*^	33.5	Chlorophyll b	462	—	—	598	647
5	34.5	Pheophytin b sp.	434	—	524	598	652
6	34.9	Pheophytin b	434	—	527	598	652
7	36.0	Pheophytin a sp.	409	503	533	609	664
8	36.6	Pheophytin a	409	506	536	610	665
9	36.8	Pheophytin a'	409	506	538	610	666
10	37.2	*β*-carotene	455	—	—	—	476
11	37.8	Pyropheophytin a	409	508	538	—	665

^*∗*^These fractions were only detected in the pigments recovered from dregs of black tea.

## Data Availability

The data used to support the findings of this study are included within the article.

## Abbreviations

LED: Light-emitting diode
HPLC: High-performance liquid chromatography
ROS: Radical oxygen species
CFU: Colony forming unit
Chl: Chlorophyll
Pheo: Pheophytin.
